# A survey of drug liking and cravings in patients using sublingual or intranasal ketamine for treatment resistant depression: A preliminary evaluation of real world addictive potential

**DOI:** 10.3389/fpsyt.2022.1016439

**Published:** 2022-11-17

**Authors:** Brittany Chubbs, Jay Wang, Shaina Archer, Carson Chrenek, Atul Khullar, Michael Wolowyk, Jennifer Swainson

**Affiliations:** ^1^Department of Psychiatry, University of Alberta, Edmonton, AB, Canada; ^2^Department of Psychiatry, University of British Columbia, Vancouver, BC, Canada; ^3^Crestwood Apothecary, Edmonton, AB, Canada

**Keywords:** ketamine, intranasal, sublingual, treatment resistant depression (TRD), addiction, abuse, drug liking, drug cravings

## Abstract

Ketamine has gained rapid popularity as a treatment option for treatment resistant depression (TRD). Though seen only in limited contexts, ketamine is a potential drug of abuse, addiction and diversion. Clinical ketamine studies to date have not systematically evaluated factors relevant to addiction risk in patients with TRD, but in treating patients with ketamine, risks of potential harms related to addiction must be considered. As clinical access to intravenous ketamine programs is limited in much of Canada, these considerations become even more important for clinicians who elect to offer patients less supervised, non-parenteral forms of ketamine treatment. This study explores factors relevant to addiction risk in a real-world sample of 33 patients with TRD currently or previously treated with sublingual (SL) or intranasal (IN) ketamine in the community. First, patients were surveyed using a Drug Liking and Craving Questionnaire (DLCQ) to assess their level of drug liking and craving for ketamine, and to screen for symptoms of a ketamine use disorder. Second, the pharmacy records of these patients were reviewed for red flags for addiction such as dose escalation or early refills. Third, surveys were administered to the treating psychiatrists of patients who had discontinued ketamine to determine if abuse concerns contributed to reason for discontinuation. Though limited to a small sample, results indicate that ketamine is not a universally liked or craved substance in patients with TRD. Prescribers of non-parenteral ketamine should monitor patients and prescribe cautiously. Factors related to addiction (as in the DLCQ) should be explored for clinicians to consider individual risk/benefit for judicious use of ketamine in patients with TRD.

## Introduction

Major depressive disorder (MDD) is a common psychiatric disorder with significant disease burden (1). As of early 2021, the global point prevalence of MDD was estimated at 3.2% (2). Approximately 15% of patients suffer from Treatment Resistant Depression (TRD), as defined by a failure to respond to two adequate trials of antidepressants from different pharmacological classes (1, 3). As such, there has been an urgent need to develop alternative treatments to target the TRD population (4).

Ketamine is a commonly used anesthetic agent and *N*-Methyl-D-aspartate (NMDA) receptor antagonist (5) which has demonstrated efficacy in treating depression at sub-anesthetic doses administered intravenously. More specifically, studies have demonstrated that a single infusion of IV ketamine, when administered at doses of 0.5–1.0 mg/kg, may provide antidepressant effects as quickly as 2 h post treatment and lasting up to 1 week, and that multiple IV ketamine infusions may extend this effect (6–11).

Intranasal (IN) esketamine, an enantiomer of ketamine, was recently approved toward the management of TRD (12). While both IV ketamine and IN esketamine represent promising treatment options for individuals with TRD, access is limited, even in urban centers (4), as both must be delivered in a healthcare setting due to monitoring requirements and concerns regarding risks of addiction or diversion (4). The United States Food and Drug Administration (FDA) has noted that subjective “liking” of a drug is the best predictor of its addictive potential (13), and IN esketamine, which has shown similar drug “liking” to ketamine in recreational drug users (14) has been placed under strict federally regulated access guidelines both in Canada and the United States. Esketamine responders are recommended for ongoing maintenance treatment (15), and though data is limited, maintenance ketamine treatment may also be necessary for some patients (11). Access to ketamine or esketamine has been further limited in the context of the COVID-19 pandemic and an associated shift toward the provision of virtual care, which has now often become patient preference.

In addressing these challenges, some physicians have opted to prescribe intranasal or sublingual (SL) forms of racemic ketamine, although evidence for use of non-IV formulations is limited to small randomized controlled trials (RCTs), anecdotal reports and case series (11). Though caution and prudence are advised, these formulations do not require the same level of supervision or monitoring, rendering them more accessible for both patients and the health care system (4). However, with increased access, the potential for abuse, misuse and addiction has been raised as a caution within several expert consensus statements on the use of ketamine for depression (9, 11, 16, 17).

Ketamine has a history as a party drug, particularly in Asian countries such as Hong Kong, Malaysia and China (18–20), and it was the most popular recreational drug of choice in Hong Kong between 2005 and 2014 (21). Despite its popularity in these countries, ketamine accounts for <1% of illicit drug use internationally, and rates of ketamine misuse are decreasing globally (22). In a ranking of overall “harm” from drugs of abuse, ketamine was ranked sixth, just behind alcohol and ahead of benzodiazepines and stimulants, which are commonly cautiously prescribed when clinically indicated in psychiatric practice (23). Another consideration is that recreational doses of ketamine are much greater than antidepressant doses. For example, one study examining 168 ketamine abusers found that they consumed a median dose of 14 g/week (typically snorted or ingested) and up to 140 g/week (24). In contrast, a meta-analysis of studies involving oral ketamine for depression included doses typically in the range of 1–2 mg/kg every 1–3 days. For further context, the largest dosing studied was 7 mg/kg TID (25), which for an 80 kg patient would translate to only 1.68 g/week.

Ketamine has a longer history of use in pain medicine, and meta-analyses from the anesthesia literature have not reported any cases of dependence or addiction to IV ketamine when used for pain management (26, 27). Similarly, two recent reviews on abuse potential for ketamine (4, 28) noted that aside from select case studies, clinical ketamine studies to date have not indicated concern for misuse, dependence, diversion, addiction in patients with TRD, and interestingly, there is emerging evidence that ketamine may be a potential treatment option for addictions (29). Very few studies, however, have included measures related to addiction or abuse in measuring side effect profile. In a systematic review of ketamine side effects, none of 20 randomized controlled trials reviewed included measures related to addiction (30). Taken together, these data make it difficult to place risk into clinical context.

As no study to date has specifically addressed risk factors for addiction in the TRD population, we set out to assess this in a real world population of patients with TRD currently or previously treated with IN or SL formulations of ketamine. To be comprehensive, we completed this study in three parts. These included (1) patient surveys to assess drug liking and craving, desire or history of using amounts of ketamine greater than prescribed, and screening questions for ketamine use disorder, (2) review of pharmacy records to look for red flags such as requests for early refills or significant dose escalation, and (3) Surveys to the psychiatrists of study participants who were no longer taking ketamine to determine whether addiction, diversion or misuse concerns had been reasons for discontinuation of treatment.

## Materials and methods

This study was conducted with approval from the University of Alberta’s Research Ethics Board. Adult patients who had filled prescriptions for compounded SL or IN ketamine at Crestwood Apothecary Pharmacy in Edmonton, AB, Canada between January 2016 and December 2020 were eligible to take part in the study. This pharmacy was selected as it has handled the majority of SL and IN ketamine prescriptions by physicians who are affiliated with the Intravenous Ketamine Program at the Misericordia Community Hospital. Patients were excluded from the study if they indicated that they had been prescribed SL or IN ketamine for indications other than major depressive episodes in the context of a bipolar spectrum disorder or Major Depressive Disorder.

The pharmacy team reviewed records of ketamine prescriptions within the specified dates to identify eligible patients, who were then contacted to explain the study and request permission to provide their contact information to the study team. Agreeable potential participants were then phoned by a member of our research team (BC) to arrange a secure online Zoom meeting. During this meeting, study information was reviewed, including all three parts of the study. Verbal informed consent was obtained and documented. This consent included all 3 parts of the study; a participant survey, review of pharmacy records, and survey by the treating psychiatrist for any patients who had discontinued ketamine.

### Participant survey

The participant survey was informed by a drug liking and craving questionnaire (DLCQ), which has been previously described and is available online (31). In the absence of a validated tool to assess addiction potential for ketamine in the psychiatric population, the DLCQ was created, based on review of the literature and recommendations for assessing abuse potential from the United States Federal Drug Administration (31). Our survey can be found in Supplementary Appendix A and included questions of drug liking, craving, desire to use more ketamine than prescribed, screening questions for a ketamine use disorder, and a place for qualitative comments. Consenting participants were sent an online link to the survey via the SurveyMonkey platform.

### Pharmacy record review

In the second part of this study, participants’ pharmacy records were reviewed (Telus Health Assyst-Rx-S software) for objective markers of ketamine misuse. Data collected included patient initials, the name of the prescribing physician, ketamine treatment route, total number of ketamine treatments, total treatment duration, starting and current/end doses, and the presence or absence of any early refills recorded and any rational for them.

### Psychiatrist survey

For participants whose ketamine had been discontinued, a third step in the study was to send the treating physician a survey to determine if concerns regarding misuse or abuse had contributed to treatment discontinuation (Supplementary Appendix B). The rationale for the questionnaire and request to complete it was sent from the study team to the physician via secure email, and again administered via the SurveyMonkey platform.

## Results

### Participation

Sixty-nine individuals were identified as eligible for participation in this research study. One was subsequently excluded due to the patient’s report that ketamine was prescribed for an indication other than depression. Fifty-seven of these potential participants were successfully contacted by telephone by the study team. Forty-four individuals consented to participate in this study, including 10 males, 1 individual who identified as non-binary, and 33 females, ranging in age from 25 to 70. Two individuals later withdrew their consent, one without providing a reason and the other reporting that they found the survey confusing and that they were too busy to continue to participate. Of the 13 individuals who declined to participate, reasons included being too busy, too unwell, or simply uninterested in hearing more about the study (*n* = 6); failing to attend the scheduled virtual meeting where informed consent was to be obtained (*n* = 6), and absence of a reliable electronic device via which to complete the survey (*n* = 1). Of the 42 consenting participants, 37 initiated survey responses and 33 surveys were completed. Thirteen individuals self-reported a diagnosis of bipolar depression and 20 self-reported unipolar depression.

### Participant questionnaire

**FIGURE 1:**
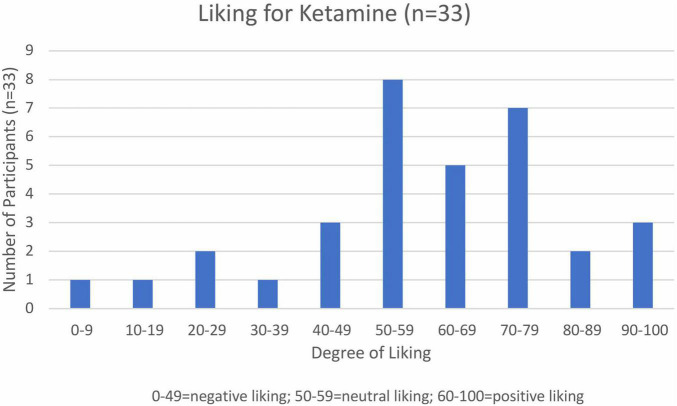
Participant ratings of “drug liking” (*n* = 33).

Participants were asked about their “liking” for ketamine on a bipolar visual analog scale (VAS) from 0–100. Scores below 50 signified a dislike for the effects of ketamine, scores between 50 and 60 were interpreted as neutral and scores above 60 were interpreted as relative “liking” for the effects of ketamine. 17/33 participants had a positive “liking” (60–100), 8/33 were neutral (50–60), and 8 reported a negative liking (0–49) (Figure 1). The overall mean “liking” of survey respondents was 57.6.

**FIGURE 2:**
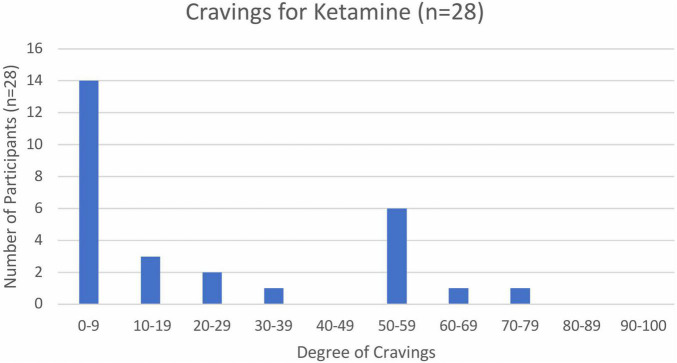
Participant ratings of “drug craving” (*n* = 28).

Cravings were measured on a unipolar VAS, with zero as “no craving” and 50 as “neutral,” while 100 was “constant desire to use ketamine.” Mean degree of cravings was 20.6, with a range from 0 to 75. Only 6 respondents rated cravings above 50, which was designated as neutral, and 4 of these were in the 51–60 range (Figure 2). The most commonly reported range was 0–9 (*n* = 14), and more than half of respondents reported cravings below 19 (Figure 2).

Drug liking and cravings have previously been determined to be markers for the risk of dependence to a substance (32–36). As the US FDA has noted drug liking as the best primary measure of abuse potential (13), and challenges have been noted in interpreting subjective reports on cravings (36), we sought to place this in better context by looking at cravings specifically in those with positive drug liking, under the clinical assumption that individuals who both “liked” ketamine and experienced cravings would be the patient population of greater risk for ketamine abuse. Of the 17 participants who reported a positive liking for ketamine (over 60), 14 responded to rate their level of craving. Of these, 5 denied cravings altogether, 3 did not specify their degree of cravings, and 9 reported variable degrees of craving ranging from 3 to 75. Two of these individuals rated their cravings above neutral (Table 1). Of the 16 individuals who had negative or neutral liking for ketamine (under 60), average cravings rating was 18.9/100; 6 had no cravings, 2 did not answer, and others ranged from 3 to 54.

**TABLE 1:** Drug cravings and substance use disorder criteria in patients with positive ketamine drug liking (*n* = 17)* (positive drug liking defined as liking > 60).

Drug liking > 60	Drug cravings (0–100)	Number of SUD criteria met
60	0	1 (time getting, using)
60	20	1 (cravings)
60	0	–
60	10	–
63	–	0
70	22	1 (tolerance)
70	3	–
70	75	1 (larger amts or longer)
72	11	0
73	65	0
73	0	0
75	0	0
80	–	1 (wanting to cut back)
80	55	1 (tolerance)
90	50	1 (tolerance)
93	–	0
100	0	–

Of the 17 individuals who “liked” the effects of ketamine, no participant met more than one substance use disorder (SUD) criterion (Table 1). Interestingly, 3 respondents in the group with neutral or negative liking for ketamine (under 60) reported 2 or more SUD criteria. Of the SUD criteria endorsed, 6 participants reported “needing more ketamine over time to get the dissociative effects you want”; 4 reported “spending a lot of time getting, using, or recovering from the use of ketamine”’; 3 reported “taking ketamine in larger amounts or longer than is prescribed”; 3 reported “cravings and urges to use ketamine”; 1 reported “wanting to cut down or stop using ketamine but being unable to do so” (for reasons other than worsening depression), and 1 reported “not managing what you should at work, home, or school because of ketamine use.” Of note, two of the four participants who reported “spending a lot of time getting, using, or recovering from the use of ketamine” clarified this by noting that it takes them hours to a full day to recover from the effects of their ketamine treatment.

**FIGURE 3:**
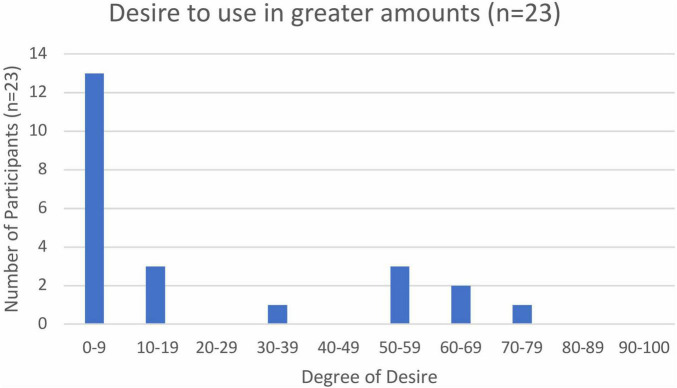
Desire to use ketamine in amounts greater than prescribed.

Ten participants failed to respond to the question regarding desire to use ketamine in doses greater than prescribed. Of those who did respond, the majority (16 of 23) reported a low “desire,” in the 0–19 range, 1 participant rated their desire in the mild-moderate range at 36, and 6 participants reported “desire” in the moderate-high (50–79) range (Figure 3). Three of 30 respondents reported that they had actually used ketamine in amounts greater than prescribed. These three individuals appeared to have similar “liking” for ketamine to the rest of the study population, but their cravings and desire to use more than prescribed were higher. Two of these individuals reported 2 SUD criteria and one endorsed 5 SUD criteria. One of these individuals commented that they had previously discontinued ketamine due to addiction. This participant was currently treated with prescription ketamine but also reported a previous history of “black market ketamine abuse.” It was not clear from this participant’s answers whether prescription, or solely illicit, ketamine had been previously discontinued due to addiction, or whether the endorsed SUD criteria related specifically to prescription ketamine or the history of illicit ketamine use.

Although 26 participants endorsed experiencing dissociative effects from their SL or IN ketamine, all survey respondents denied having used their prescription ketamine to “get high.” No participant had shared their prescription ketamine with others, but two respondents endorsed having “considered” sharing their ketamine. These individuals specified that the reason was so that their loved ones would know what they experience when they take it. One respondent reported that their prescription ketamine had been stolen and specified in the written comments that this was by a family member who also suffered from depression. Several other participants described taking measures such as not telling others they are on ketamine and/or keeping their ketamine locked up to reduce the risk that this medication would be stolen. Finally, of the 33 participants who responded to the question regarding the presence of dissociative side effects, 26 reported that they do experience dissociative effects from ketamine, whilst 7 responded that they do not.

On qualitative review of comments that participants submitted as part of their survey responses, several themes emerged. Perhaps most notably, participants held strong yet opposing views regarding the dissociative side effects from ketamine. For example, six participants used terms such as “relaxing” and “peaceful” to describe their experience of dissociative side effects, whilst six others described such experiences as “unnerving” and “terrifying.” Of further interest, five participants alluded to some “wearing off” of dissociative effects with continued use, six participants suggested that they could “see how” ketamine could become addictive (and cited the “relaxation and peaceful feeling” they experienced following ketamine administration and the “wanting/craving” that can be experienced between treatments to substantiate this concern), and three participants commented that they had not noticed any signs of addiction within their experience of using prescription ketamine. Further, two participants reported taking their ketamine at a dose or frequency lower than prescribed.

### Pharmacy record review

Pharmacy records were reviewed for the 42 consenting participants. Of these, 21 participants had ever been prescribed IN ketamine and 37 had ever been prescribed SL ketamine, indicating that some participants had had trials of both. The starting dose of IN for all patients was 100 mg, and it was increased to 150 mg in 5 patients and 140 mg in 1 patient. All others were maintained at 100 mg. The average duration of treatment with IN ketamine was 41.2 weeks, with a range of 5–243 weeks, and 5–389 total treatments. For SL ketamine, the starting doses ranged from 50 to 200 mg with most initial doses at 100 or 150 mg. Fifteen of 37 patients had dose increases to a maximum of 300 mg, while 3 patients had dose decreases. The other 19 participants remained at consistent dosing. The average duration of treatment with SL ketamine was 79.3 weeks, with a range of 4–570 weeks, and 7–990 total treatments. Finally, there were 5 documented early refill requests amongst 5 different participants. Of these, 1 request was to accommodate a pharmacy closure; 1 to accommodate a patient’s holiday; 1 “due to nasal spray liquid not lasting until the estimated time of supply”; and 2 for reasons which were not documented in the pharmacy records.

### Physician surveys

Nine physicians were identified as having prescribed ketamine to 15 participants who were “previously treated” with ketamine. Physician surveys were completed for all 15 patients. These surveys indicated no addiction concerns; there were no instances of discontinued ketamine prescribing due to addiction or misuse concerns. Similarly, there were no concerns regarding ketamine diversion and no physicians were aware of participants developing dependency to another recreational substance during their treatment with ketamine.

## Discussion

Although this study is descriptive in nature and reports on a small cohort of patients, results suggest that patients prescribed SL or IN ketamine for depression are not universally at risk of drug misuse abuse or diversion. Of the 57 potential participants contacted, only 42 consented and only 33 fully completed surveys. We would posit that this is partially due to the retrospective nature of the survey and the fact it was conducted completely virtually (due to COVID-19), but the possibility for significant response bias exists. Patients with more concerns for addiction/dependence may not have consented to participate or failed to complete their survey. Respondents who were still actively using ketamine treatment may have also under-reported symptoms/signs suggestive of addiction due to concern of having their ketamine prescription changed or discontinued.

Another potential limiting factor was that prospective participants were identified via records from a single pharmacy. This pharmacy has been the primary pharmacy in Edmonton to compound ketamine for psychiatrists who work within Edmonton’s ketamine programs, and while participants were the patients of 14 different physicians, data collection from this pharmacy alone does limit the pool of prospective participants and prescribers.

The survey itself did not separate experiences with IN or SL ketamine, as many patients had had trials of both. Questions were asked about ketamine in general, and many of these patients had also had previous courses of IV ketamine. In considering abuse/addiction potential, differing pharmacokinetics of the various formulations may impact factors such as liking and craving. Future versions of the survey should also clarify the craving continuum on the VAS to better separate and attribute meaning to those ratings between 1 and 50, as in the current form, they were difficult to interpret. The physician survey was also limited as it was based on clinical judgment only, rather than standardized patient assessment.

Despite multiple limitations, to our knowledge, this was the first study to specifically attempt to assess abuse/addiction potential of ketamine in patients treated with ketamine for TRD. While true level of risk remains unclear, results of this study suggest that prescribing of SL and IN ketamine for TRD need not be viewed with strict prohibition due to addiction concerns, but instead placed within appropriate clinical context of risk/benefit on an individual basis. While this study had a high risk of reporting bias, it remains reassuring that ketamine is not a universally highly liked or craved substance among patient with TRD. Patients surveyed were not using it to “get high” and very few patients desired to use more than prescribed. Ketamine appears not dissimilar to other drugs in psychiatry, such as stimulants or hypnotics, which carry both potential for abuse and for therapeutic benefit for the appropriate patient. Several authors in our group have previously made recommendations for judicious prescribing of non-parenteral ketamine (4), including appropriate patient selection and prescribing considerations. Ketamine prescribers would be advised to use a tool similar to the DLCQ which, though unvalidated and could be improved upon, is a simple tool that can be found online (31). Use of the DLCQ or a similar tool could allow prescribers to routinely monitor patients for signs of drug “liking,” “craving,” and “desire.” Prescribers should also ask patients about misuse of their ketamine, and screen for criteria of a ketamine use disorder. As seen in this study, this data alone is not sufficient due to multiple confounding factors but can be collected as an opening to further discussion to clinically assess abuse/addiction risks for each patient. In the absence of a validated tool, DLCQ or similar scale to assess drug liking could be used in clinical studies to better evaluate and document risk factors related to addiction and misuse of ketamine and esketamine. The DLCQ is currently in use in a real-world study on efficacy of esketamine (37). Though this is a small sample size with qualitative data reporting, Table 1 would appear to indicate that drug liking is not always associated with craving, and that craving level is quite variable (3–75 range). On review of survey responses, interpretation of cravings rated between zero and 50 is a significant limiting factor in interpretation of our study. In the future, we would suggest adjusting descriptors on the scale to describe intensity and/or frequency along the continuum. We would posit that the largest risk of ketamine misuse or abuse would be in individuals with a high drug liking who also experienced significant cravings for the substance. While there was a subset of patients who did “like” and “crave” the drug, this was not universal. The ketamine treatment experience for TRD appears to be not universally pleasant, enjoyable or desired and this dovetails with many of the authors’ significant clinical experience.

As part of this study, we screened for SUD criteria for ketamine. The two most commonly reported SUD criteria were “needing more ketamine over time to get the dissociative effects you want” (i.e., tolerance) and “spending a lot of time getting, using, or recovering from the use of ketamine.” In the case of ketamine treatment, tolerance to dissociative symptoms is common and even expected. Clinical experience also indicates that patients may feel more tired the rest of the day following treatment, have side effects such as fatigue or headache, or find the dissociative experience to be emotional. In addition, patients are restricted from driving until the following day after ketamine administration, and thus limited with respect to their usual functioning. As these are common and expected effects following ketamine treatment, they may not be concerning for a SUD. In viewing results in this context, individual positive responses for these one or two SUD seem to carry little relevance in assessing overall risk level, and cannot be interpreted as indicative of a ketamine use disorder in the absence of further data. This is a significant limitation in interpreting this data. Future studies should provide a clear preamble to questions screening for SUD to specify that these statements would NOT relate to any desired dissociative effects for the expected purpose of improved antidepressant efficacy, nor would they relate time required to recover from a standard ketamine treatment.

As no participant endorsed a desire to use ketamine to get “high,” we would posit that the most frequent reason a patient would desire to use more ketamine than prescribed would be an expectation that more ketamine could further improve their depression. The patients on maintenance ketamine in this study were a population of highly treatment resistant individuals, and it has been the authors’ experience that when individuals with TRD respond to ketamine, much hope is placed in this medication. As part of informed consent for clinical treatment, these patients have also generally been informed by their psychiatrist about the lack of data regarding dosing and duration of treatment for IN and SL ketamine, and this may lead patients to wonder about using more than currently prescribed. Any future studies should include follow up questions as to the reasons why an individual would seek to use extra ketamine to better elucidate level of risk.

Six participants in our study reported needing to use more ketamine over time to achieve a desired dissociative effect, but large dose increases were not seen despite long durations of treatment. Due to lack of data to guide dosing of these formulations, dose increases likely reflect clinical dosing titration. Further, these 6 individuals were neutral or negative regarding their experience of ketamine, so in this context, this finding likely reflects only tachyphylaxis to dissociation during ongoing ketamine treatment, rather than serving as a red flag for abuse potential. Even if dissociation is not a positive experience, continued dissociative experiences may be desired if patients misattribute the presence of dissociation as an indication that ketamine is “working” for their depression. Though psychedelic psychotherapies, including ketamine psychotherapy, are gaining popularity and rely on dissociation as part of the therapeutic effect, the patients in this study were receiving ketamine only as a part of a pharmacotherapy regimen. Psychoeducation prior to treatment with ketamine should include the concept that dissociative experiences are variable and not necessarily correlated to the antidepressant effects when using ketamine as a pharmacotherapeutic tool. Patient desire to use excess medication should be assessed on an ongoing basis in patients on maintenance ketamine, and reasons for wanting to use more need to be explored by treating physicians to best assess level of risk.

Though no participant endorsed a desire to use their ketamine to “get high,” one participant did report a history of illicit ketamine use. Interestingly, in this case, illicit ketamine use in the past did not translate to a desire to abuse prescribed ketamine. In this context, we query whether this individual had used illicit ketamine to self-medicate prior to being prescribed ketamine. Limited access to ketamine programs may stimulate illicit ketamine use by a subset of patients attempting to self medicate a depressive illness, and this uncontrolled use should be strongly discouraged. Increased access to appropriately prescribed SL or IN ketamine may help prevent this uncontrolled use.

Future research assessing ketamine for mental health indications should include measures of addictive potential to further elucidate potential risks so clinicians can better evaluate risks and benefits of treatment.

## Data availability statement

The datasets presented in this article are not readily available because Permissions were not obtained from ethics to share data. Requests to access the datasets should be directed to JS, Jennifer.swainson@ualberta.ca.

## Ethics statement

The studies involving human participants were reviewed and approved by University of Alberta Health Research Ethics Board. The patients/participants provided verbal informed consent to participate in this study, which was documented by a study investigator over a virtual meeting.

## Author contributions

JS, JW, BC, SA, CC, and AK contributed to study design and interpretation of results. SA, JW, and BC contributed to the ethics proposal. BC and MW collected the data. BC wrote the first draft of the manuscript, under supervision and contribution from JS. BC and JS prepared the manuscript revision. All authors reviewed and approved the final manuscript for publication.
