# Supplementary material for: A survey of drug liking and cravings in patients using sublingual or intranasal ketamine for treatment resistant depression: A preliminary evaluation of real world addictive potential
Source: Front Psychiatry. 2022 Nov 17;13:1016439. doi: 10.3389/fpsyt.2022.1016439 (PMC9714431; doi:10.3389/fpsyt.2022.1016439)
Supplement: Supplementary file 1 [file Data_Sheet_1.pdf]

Date:

APPENDIX : Patient Questionnaire

Pt Initial:______________________

Pt Year of Birth:______________________

Pt Study ID:__________________________

Instruction

In this questionnaire, you will be asked to provide responses according to the degree of your experience. To do so, you will be asked to put a vertical line on a scale from 0 – 10, which is meant to represent the degree to which you agree or disagree with the question. In addition, you will be asked to put your answer in the box to the right of the scale. An example is provided below. Please read the scale carefully to understand what the numbers on the scales represent.

Sample Response:

Overall, my liking for vanilla ice cream is: (0 = strong dislike, 5 = neutral, 10 = strong liking)

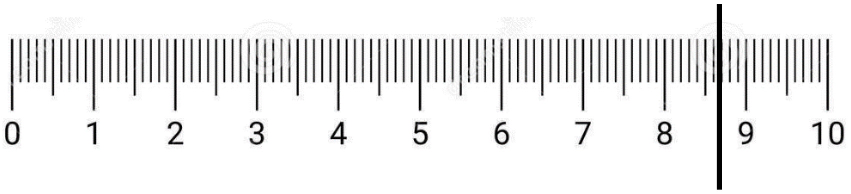

8.7

Please proceed to page 2 -5 to complete the questionnaire.

Date:

What are your initials?

What is your gender?

- ○ Female
- ○ Male
- ○ Other (please specify)

Most people who are prescribed ketamine have a diagnosis of depression. Which of the following are you using ketamine for?

- ○ Depression
- ○ Bipolar depression
- ○ Don't know

Likeability for Ketamine

"Liking" can be understood as the degree one looks forward to the positive psychoactive effects of a substance.[1,2] Examples of these effects can include experiences such as feeling "high", feeling detached from one's body, feelings of floating, feelings of wellbeing, experience of strong emotions, or increased sensitivity to the senses such as light or sound.

Please assess your degree of likeability for ketamine per the following questions by putting one vertical line on each of the following scale.

a.) Overall, my liking for ketamine is:
(0 = strong dislike; 5 = neutral; 10 = strong liking)

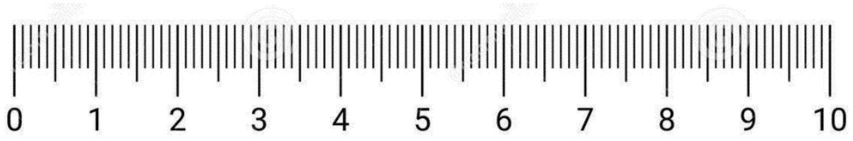

Craving for ketamine

Date:

“Craving” for a substance can be understood as an intense desire and motivation to acquire and to use a drug. Please assess your degree of craving for ketamine per the following questions by putting one vertical line on each of the following scale.

a.) Overall, how much do you crave ketamine treatments for effects other than its antidepressant effects?
(0 = absolutely no craving, 5 = neutral, 10 = constantly craving)

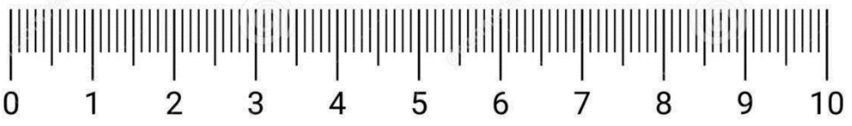

b.) How much do you desire to use ketamine in greater amounts or higher frequency than your psychiatrist is prescribing? This does not need to mean you have done so, but that you experience desire in between treatments to experience the psychoactive effects of ketamine.
(0 = absolutely no craving, 5 = neutral, 10 = very strong craving)

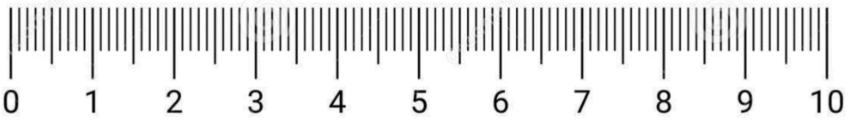

Ketamine is a substance that potentially can become problematic. Have you experienced any of the following?

☐ Taking ketamine in larger amounts or longer than is prescribed

☐ Wanting to cut down or stop using ketamine but being unable to do so (for reasons other than worsening depression)

Date:

☐ Spending a lot of time getting, using, or recovering from the use of ketamine
☐ Cravings and urges to use ketamine
☐ Not managing what you should at work, home, or school because of ketamine use
☐ Continuing to use ketamine even when it causes problems in relationships
☐ Giving up important social, work, or recreational activities because of ketamine use
☐ Using ketamine even when it puts you in dangerous situations (e.g. using ketamine and driving when advised not to)
☐ Continuing to use ketamine when you know it's causing problems with your help (e.g. having side effects but continuing to use ketamine)
☐ Needing more ketamine over time to get the dissociative effects you want
☐ Development of withdrawal symptoms which can be relieved by taking more ketamine
Comments?

When you take ketamine do you experience dissociative side effects (e.g. feeling high, perceptual disturbances, feeling outside of body or floating, feeling that you or things around you are not real)

○ Yes
○ No
Comments?

Have you ever used ketamine in greater amounts or more often than prescribed by your psychiatrist?

○ Yes
○ No
Comments?

Have you ever obtained illicit (street) ketamine other than what is prescribed to you?

○ Yes
○ No
Comments?

Have you ever used your ketamine to get high?

○ Yes

Date:

◯ No
Comments?

Have you ever shared your ketamine with someone else?

◯ Yes
◯ No
Comments?

Have you ever considered sharing your ketamine with someone else?

◯ Yes
◯ No
Comments?

If you have discontinued ketamine, was it due to addiction?

◯ Yes
◯ No
Comments?

Have you ever had ketamine stolen from you?

◯ Yes
◯ No
Comments?

This study is examining potential for addiction to ketamine, when used to treat depression. As a patient who has experienced ketamine treatments, do you have any comments to share with the study team to further describe the psychoactive effects of ketamine and any desire you have to experience these effects specifically?
